# 4-(4-Fluoro­phen­yl)-1-(4-nitro­phen­yl)-3-(pyridin-4-yl)-1*H*-pyrazol-5-amine

**DOI:** 10.1107/S1600536812004102

**Published:** 2012-02-10

**Authors:** Bassam Abu Thaher, Pierre Koch, Dieter Schollmeyer, Stefan Laufer

**Affiliations:** a Faculty of Science, Chemistry Department, Islamic University of Gaza, Gaza Strip, Palestinian Territories; b Institute of Pharmacy, Department of Pharmaceutical and Medicinal Chemistry, Eberhard Karls University Tübingen, Auf der Morgenstelle 8, 72076 Tübingen, Germany; c Department of Organic Chemistry, Johannes Gutenberg University Mainz, Duesbergweg 10-14, D-55099 Mainz, Germany

## Abstract

In the crystal structure of the title compound, C_20_H_14_FN_5_O_2_, the pyrazole ring forms dihedral angles of 59.3 (2), 25.6 (2) and 46.0 (2)° with the directly attached 4-fluoro­phenyl, pyridine and nitro­phenyl rings, respectively. The crystal packing is characterized by inter­molecular N—H⋯N and N—H⋯O hydrogen bonds.

## Related literature

For p38α MAP kinase inhibitors having a vicinal 4-fluoro­phen­yl/pyridin-4-yl system connected to a five-membered heterocyclic core, see: Abu Thaher *et al.* (2009 ▶); Peifer *et al.* (2006 ▶). For inhibitory activity and preparation of the title compound, see: Abu Thaher *et al.* (2012 ▶).
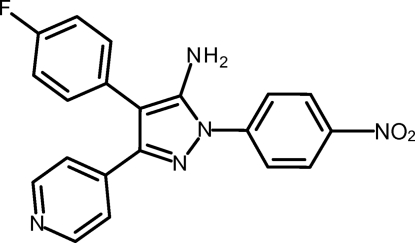


## Experimental

### 

#### Crystal data

C_20_H_14_FN_5_O_2_
*M* *_r_* = 375.36
Triclinic, 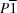

*a* = 8.5088 (14) Å
*b* = 9.8797 (11) Å
*c* = 10.4264 (14) Å
α = 79.906 (10)°
β = 78.764 (10)°
γ = 86.245 (9)°
*V* = 845.9 (2) Å^3^
*Z* = 2
Cu *K*α radiation
μ = 0.89 mm^−1^
*T* = 193 K
0.35 × 0.35 × 0.20 mm

#### Data collection

Enraf–Nonius CAD-4 diffractometer
3416 measured reflections
3185 independent reflections
2835 reflections with *I* > 2σ(*I*)
*R*_int_ = 0.020
3 standard reflections every 60 min intensity decay: 2%

#### Refinement

*R*[*F* ^2^ > 2σ(*F* ^2^)] = 0.070
*wR*(*F* ^2^) = 0.247
*S* = 1.18
3185 reflections
254 parameters
H-atom parameters constrained
Δρ_max_ = 0.49 e Å^−3^
Δρ_min_ = −0.73 e Å^−3^

### 

Data collection: *CAD-4 Software* (Enraf–Nonius, 1989 ▶); cell refinement: *CAD-4 Software*; data reduction: *CORINC* (Dräger & Gattow, 1971 ▶); program(s) used to solve structure: *SIR97* (Altomare *et al.*, 1999 ▶); program(s) used to refine structure: *SHELXL97* (Sheldrick, 2008 ▶); molecular graphics: *PLATON* (Spek, 2009 ▶); software used to prepare material for publication: *PLATON*.

## Supplementary Material

Crystal structure: contains datablock(s) I, global. DOI: 10.1107/S1600536812004102/bt5809sup1.cif

Structure factors: contains datablock(s) I. DOI: 10.1107/S1600536812004102/bt5809Isup2.hkl

Supplementary material file. DOI: 10.1107/S1600536812004102/bt5809Isup3.cml

Additional supplementary materials:  crystallographic information; 3D view; checkCIF report

## Figures and Tables

**Table 1:** Hydrogen-bond geometry (Å, °)

*D*—H⋯*A*	*D*—H	H⋯*A*	*D*⋯*A*	*D*—H⋯*A*
N6—H6*A*⋯O14^i^	0.91	2.29	3.149 (4)	158
N6—H6*B*⋯N26^ii^	0.87	2.19	2.985 (3)	151

Symmetry codes: (i) 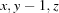; (ii) 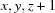.

**Fig. 1.**
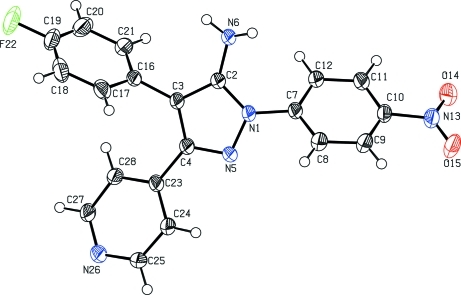
View of the title compound. Displacement ellipsoids are drawn at the 50% probability level. H atoms are depicted as circles of arbitrary size.

**Fig. 2.**
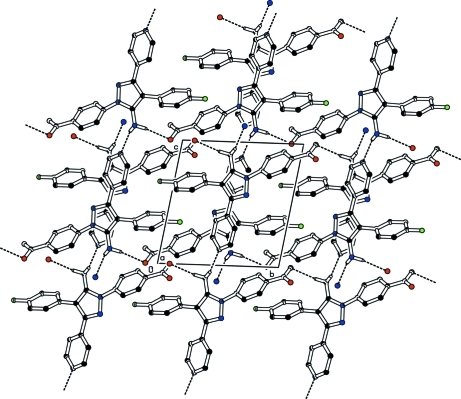
Crystal structure of the title compound with view along the a-axis (hydrogen bonding is shown with dashed lines).

## supplementary crystallographic information

### Comment

Compounds having a vicinal 4-fluorophenyl/pyridin-4-yl system connected to a five-membered heterocyclic core have been considered to be potential p38α MAP kinase inhibitors (Abu Thaher *et al.* 2009, Peifer *et al.* 2006). Recently, we showed that the regioisomeric switch from 3-(4-fluorophenyl)-4-(pyridin-4-yl)-1-(aryl)-1*H*-pyrazol-5-amine to 4-(4-fluorophenyl)-3-(pyridin-4-yl)-1-(aryl)-1*H*-pyrazol-5-amine completely changed the inhibitory profile from p38α MAP kinase to kinases releant in cancer (Abu Thaher *et al.* 2012).

In the crystal structure of the title compound (Fig. 1), the pyrazole ring forms dihedral angels of 59.3 (2)°, 25.6 (2)° and 46.0 (2)° with the 4-fluorophenyl, pyridine and nitrophenyl rings, respectively. The 4-fluorophenyl ring encloses dihedral angels of 59.3 (2)° and 17.5 (2)° toward the pyridine and 4-nitrophenyl rings, respectively. The pyridine ring is orientated at a dihedral angle of 42.4 (2)° toward the nitrophenyl ring.

The crystal packing (Fig. 2) shows that the amino function acts as a hydrogen bond donor of two intermolecular hydrogen bonds - one to the nitrogen atom (N26) of the pyridine ring and another one to one oxygen atom (O14) of the nitro group of two different molecules. The length of the hydrogen bonds is 2.19 Å and 2.29 Å, respectively (Table 1). The two hydrogen bonds result in a two dimensional network parallel to the b-c-plan.

### Experimental

20 mmol of LDA was added to 30 ml of dry THF in a three neck flask and cooled to 195 K. 14 mmol of 4-fluorophenylacetonitril in 10 ml THF was added dropwise and the reaction mixture was stirred for 45 min. 5 mmol of *N*-(4-nitrophenyl)-4-pyridinecarbohydrazonoyl chloride was added slowly to the reaction and stirring was continued for 1 h. After warming to 293 K, 50 ml of water was added to the reaction mixture and extracted with ethyl acetate (2x 50 ml). The organic layer was dried over Na_2_SO_4_. The solvent was removed under redued pressure to about 5 ml and the pure product precipitated. Yield: 36%. Recrystallization from THF/diethylether resulted in crystals suitable for X-ray.

### Refinement

Hydrogen atoms attached to carbons were placed at calculated positions with C—H = 0.95 Å (aromatic) or 0.98–0.99 Å (*sp*^3^ C-atom). H atoms bonded to N were located in a difference map and constrained to this position. All H atoms were refined in the riding-model approximation with isotropic displacement parameters (set at 1.2–1.5 times of the *U*_eq_ of the parent atom).

### Crystal data

**Table:**

C_20_H_14_FN_5_O_2_	*Z* = 2
*M**_r_* = 375.36	*F*(000) = 388
Triclinic, *P*1	*D*_x_ = 1.474 Mg m^−^^3^
Hall symbol: -P 1	Cu *K*α radiation, λ = 1.54178 Å
*a* = 8.5088 (14) Å	Cell parameters from 25 reflections
*b* = 9.8797 (11) Å	θ = 65–69°
*c* = 10.4264 (14) Å	µ = 0.89 mm^−^^1^
α = 79.906 (10)°	*T* = 193 K
β = 78.764 (10)°	Block, brown
γ = 86.245 (9)°	0.35 × 0.35 × 0.20 mm
*V* = 845.9 (2) Å^3^	

### Data collection

**Table:**

Enraf–Nonius CAD-4 diffractometer	*R*_int_ = 0.020
Radiation source: rotating anode	θ_max_ = 69.8°, θ_min_ = 4.4°
Graphite monochromator	*h* = −10→0
ω/2θ scans	*k* = −12→12
3416 measured reflections	*l* = −12→12
3185 independent reflections	3 standard reflections every 60 min
2835 reflections with *I* > 2σ(*I*)	intensity decay: 2%

### Refinement

**Table:**

Refinement on *F*^2^	Secondary atom site location: difference Fourier map
Least-squares matrix: full	Hydrogen site location: inferred from neighbouring sites
*R*[*F*^2^ > 2σ(*F*^2^)] = 0.070	H-atom parameters constrained
*wR*(*F*^2^) = 0.247	*w* = 1/[σ^2^(*F*_o_^2^) + (0.1328*P*)^2^ + 1.0029*P*] where *P* = (*F*_o_^2^ + 2*F*_c_^2^)/3
*S* = 1.18	(Δ/σ)_max_ < 0.001
3185 reflections	Δρ_max_ = 0.49 e Å^−^^3^
254 parameters	Δρ_min_ = −0.73 e Å^−^^3^
0 restraints	Extinction correction: *SHELXL97* (Sheldrick, 2008), Fc^*^=kFc[1+0.001xFc^2^λ^3^/sin(2θ)]^-1/4^
Primary atom site location: structure-invariant direct methods	Extinction coefficient: 0.056 (6)

### Special details

**Table:**

Geometry. All e.s.d.'s (except the e.s.d. in the dihedral angle between two l.s. planes) are estimated using the full covariance matrix. The cell e.s.d.'s are taken into account individually in the estimation of e.s.d.'s in distances, angles and torsion angles; correlations between e.s.d.'s in cell parameters are only used when they are defined by crystal symmetry. An approximate (isotropic) treatment of cell e.s.d.'s is used for estimating e.s.d.'s involving l.s. planes.
Refinement. Refinement of *F*^2^ against ALL reflections. The weighted *R*-factor *wR* and goodness of fit *S* are based on *F*^2^, conventional *R*-factors *R* are based on *F*, with *F* set to zero for negative *F*^2^. The threshold expression of *F*^2^ > σ(*F*^2^) is used only for calculating *R*-factors(gt) *etc*. and is not relevant to the choice of reflections for refinement. *R*-factors based on *F*^2^ are statistically about twice as large as those based on *F*, and *R*- factors based on ALL data will be even larger.

### Fractional atomic coordinates and isotropic or equivalent isotropic displacement parameters (Å^2^)

**Table:**

	*x*	*y*	*z*	*U*_iso_*/*U*_eq_	
N1	0.2765 (3)	0.5908 (2)	0.7116 (2)	0.0313 (6)	
C2	0.2170 (4)	0.4635 (3)	0.7619 (3)	0.0296 (7)	
C3	0.2085 (4)	0.4005 (3)	0.6544 (3)	0.0303 (7)	
C4	0.2649 (4)	0.5007 (3)	0.5421 (3)	0.0309 (7)	
N5	0.3086 (3)	0.6147 (3)	0.5750 (2)	0.0325 (6)	
N6	0.1761 (3)	0.4154 (3)	0.8957 (2)	0.0339 (6)	
H6A	0.1666	0.3231	0.9181	0.051*	
H6B	0.2382	0.4442	0.9420	0.051*	
C7	0.3021 (4)	0.6985 (3)	0.7779 (3)	0.0306 (7)	
C8	0.4406 (4)	0.7722 (3)	0.7327 (3)	0.0385 (8)	
H8	0.5222	0.7431	0.6661	0.046*	
C9	0.4599 (4)	0.8882 (3)	0.7848 (3)	0.0407 (8)	
H9	0.5546	0.9394	0.7556	0.049*	
C10	0.3373 (4)	0.9277 (3)	0.8805 (3)	0.0365 (8)	
C11	0.2029 (4)	0.8520 (3)	0.9310 (3)	0.0369 (7)	
H11	0.1230	0.8802	0.9992	0.044*	
C12	0.1854 (4)	0.7340 (3)	0.8809 (3)	0.0338 (7)	
H12	0.0952	0.6783	0.9163	0.041*	
N13	0.3484 (4)	1.0589 (3)	0.9244 (3)	0.0436 (7)	
O14	0.2312 (4)	1.1002 (2)	0.9998 (3)	0.0565 (8)	
O15	0.4705 (4)	1.1239 (3)	0.8826 (3)	0.0571 (8)	
C16	0.1488 (4)	0.2617 (3)	0.6615 (3)	0.0322 (7)	
C17	0.2460 (5)	0.1614 (3)	0.6016 (3)	0.0402 (8)	
H17	0.3543	0.1802	0.5616	0.048*	
C18	0.1847 (6)	0.0344 (4)	0.6006 (4)	0.0535 (11)	
H18	0.2493	−0.0331	0.5583	0.064*	
C19	0.0299 (6)	0.0088 (4)	0.6616 (4)	0.0530 (11)	
C20	−0.0680 (5)	0.1035 (4)	0.7234 (4)	0.0537 (10)	
H20	−0.1751	0.0827	0.7655	0.064*	
C21	−0.0064 (4)	0.2304 (3)	0.7228 (3)	0.0410 (8)	
H21	−0.0724	0.2970	0.7654	0.049*	
F22	−0.0294 (4)	−0.1151 (2)	0.6613 (3)	0.0839 (11)	
C23	0.2733 (4)	0.4946 (3)	0.4002 (3)	0.0311 (7)	
C24	0.3791 (4)	0.5746 (3)	0.3017 (3)	0.0325 (7)	
H24	0.4492	0.6336	0.3246	0.039*	
C25	0.3810 (4)	0.5674 (3)	0.1697 (3)	0.0351 (7)	
H25	0.4530	0.6237	0.1038	0.042*	
N26	0.2871 (4)	0.4856 (3)	0.1299 (2)	0.0377 (7)	
C27	0.1860 (4)	0.4099 (3)	0.2248 (3)	0.0380 (8)	
H27	0.1185	0.3511	0.1985	0.046*	
C28	0.1722 (4)	0.4108 (3)	0.3597 (3)	0.0358 (7)	
H28	0.0960	0.3559	0.4228	0.043*	

### Atomic displacement parameters (Å^2^)

**Table:**

	*U*^11^	*U*^22^	*U*^33^	*U*^12^	*U*^13^	*U*^23^
N1	0.0511 (15)	0.0246 (12)	0.0214 (12)	−0.0101 (10)	−0.0109 (10)	−0.0043 (9)
C2	0.0429 (15)	0.0256 (14)	0.0222 (13)	−0.0076 (11)	−0.0086 (11)	−0.0039 (11)
C3	0.0468 (16)	0.0247 (14)	0.0228 (13)	−0.0090 (12)	−0.0129 (12)	−0.0028 (11)
C4	0.0462 (16)	0.0268 (14)	0.0226 (14)	−0.0093 (12)	−0.0126 (12)	−0.0026 (11)
N5	0.0520 (15)	0.0280 (12)	0.0194 (11)	−0.0121 (10)	−0.0087 (10)	−0.0029 (9)
N6	0.0572 (16)	0.0265 (12)	0.0207 (12)	−0.0128 (11)	−0.0116 (11)	−0.0023 (9)
C7	0.0497 (17)	0.0234 (14)	0.0220 (13)	−0.0074 (12)	−0.0134 (12)	−0.0027 (10)
C8	0.0541 (19)	0.0316 (16)	0.0323 (16)	−0.0127 (14)	−0.0101 (14)	−0.0053 (12)
C9	0.0567 (19)	0.0307 (16)	0.0373 (17)	−0.0164 (14)	−0.0114 (14)	−0.0044 (13)
C10	0.063 (2)	0.0234 (15)	0.0271 (14)	−0.0100 (13)	−0.0179 (14)	−0.0027 (11)
C11	0.060 (2)	0.0285 (15)	0.0250 (14)	−0.0076 (13)	−0.0133 (13)	−0.0042 (11)
C12	0.0519 (18)	0.0271 (15)	0.0243 (14)	−0.0106 (13)	−0.0097 (12)	−0.0033 (11)
N13	0.076 (2)	0.0256 (13)	0.0334 (14)	−0.0117 (13)	−0.0178 (14)	−0.0038 (11)
O14	0.094 (2)	0.0309 (13)	0.0451 (14)	−0.0082 (13)	−0.0039 (14)	−0.0144 (11)
O15	0.084 (2)	0.0341 (13)	0.0586 (17)	−0.0257 (13)	−0.0204 (14)	−0.0071 (12)
C16	0.0545 (18)	0.0257 (14)	0.0198 (13)	−0.0108 (12)	−0.0149 (12)	−0.0005 (10)
C17	0.066 (2)	0.0311 (16)	0.0273 (15)	0.0001 (14)	−0.0170 (14)	−0.0058 (12)
C18	0.104 (3)	0.0278 (16)	0.0387 (18)	0.0041 (18)	−0.036 (2)	−0.0099 (14)
C19	0.099 (3)	0.0296 (17)	0.0393 (18)	−0.0258 (18)	−0.037 (2)	0.0055 (14)
C20	0.078 (3)	0.048 (2)	0.0398 (18)	−0.0335 (19)	−0.0242 (18)	0.0077 (16)
C21	0.058 (2)	0.0366 (17)	0.0298 (15)	−0.0182 (15)	−0.0110 (14)	−0.0009 (13)
F22	0.162 (3)	0.0346 (12)	0.0725 (17)	−0.0424 (15)	−0.0671 (19)	0.0075 (11)
C23	0.0493 (17)	0.0246 (14)	0.0227 (14)	−0.0061 (12)	−0.0139 (12)	−0.0027 (11)
C24	0.0458 (16)	0.0280 (14)	0.0264 (14)	−0.0110 (12)	−0.0101 (12)	−0.0041 (11)
C25	0.0506 (18)	0.0321 (15)	0.0231 (14)	−0.0133 (13)	−0.0074 (12)	−0.0006 (11)
N26	0.0587 (17)	0.0333 (14)	0.0249 (12)	−0.0115 (12)	−0.0161 (11)	−0.0024 (10)
C27	0.0579 (19)	0.0326 (16)	0.0286 (15)	−0.0163 (14)	−0.0184 (14)	−0.0023 (12)
C28	0.0558 (18)	0.0292 (15)	0.0252 (14)	−0.0151 (13)	−0.0155 (13)	0.0018 (11)

### Geometric parameters (Å, º)

**Table:**

N1—C2	1.365 (4)	N13—O14	1.238 (4)
N1—N5	1.378 (3)	C16—C21	1.379 (5)
N1—C7	1.416 (3)	C16—C17	1.401 (5)
C2—N6	1.376 (4)	C17—C18	1.393 (5)
C2—C3	1.390 (4)	C17—H17	0.9500
C3—C4	1.420 (4)	C18—C19	1.366 (6)
C3—C16	1.478 (4)	C18—H18	0.9500
C4—N5	1.329 (4)	C19—F22	1.355 (4)
C4—C23	1.479 (4)	C19—C20	1.372 (7)
N6—H6A	0.9055	C20—C21	1.389 (5)
N6—H6B	0.8721	C20—H20	0.9500
C7—C8	1.384 (4)	C21—H21	0.9500
C7—C12	1.390 (4)	C23—C24	1.392 (4)
C8—C9	1.383 (4)	C23—C28	1.400 (4)
C8—H8	0.9500	C24—C25	1.387 (4)
C9—C10	1.383 (5)	C24—H24	0.9500
C9—H9	0.9500	C25—N26	1.340 (4)
C10—C11	1.372 (5)	C25—H25	0.9500
C10—N13	1.463 (4)	N26—C27	1.331 (4)
C11—C12	1.386 (4)	C27—C28	1.390 (4)
C11—H11	0.9500	C27—H27	0.9500
C12—H12	0.9500	C28—H28	0.9500
N13—O15	1.223 (4)		
			
C2—N1—N5	112.1 (2)	O14—N13—C10	118.0 (3)
C2—N1—C7	129.9 (2)	C21—C16—C17	118.8 (3)
N5—N1—C7	117.9 (2)	C21—C16—C3	120.4 (3)
N1—C2—N6	123.1 (2)	C17—C16—C3	120.8 (3)
N1—C2—C3	106.9 (2)	C18—C17—C16	120.3 (4)
N6—C2—C3	130.0 (3)	C18—C17—H17	119.8
C2—C3—C4	104.2 (2)	C16—C17—H17	119.8
C2—C3—C16	125.9 (3)	C19—C18—C17	118.7 (4)
C4—C3—C16	129.9 (2)	C19—C18—H18	120.7
N5—C4—C3	112.7 (2)	C17—C18—H18	120.7
N5—C4—C23	118.7 (2)	F22—C19—C18	118.7 (4)
C3—C4—C23	128.6 (3)	F22—C19—C20	118.7 (4)
C4—N5—N1	104.1 (2)	C18—C19—C20	122.6 (3)
C2—N6—H6A	115.2	C19—C20—C21	118.3 (4)
C2—N6—H6B	113.1	C19—C20—H20	120.9
H6A—N6—H6B	110.1	C21—C20—H20	120.9
C8—C7—C12	121.2 (3)	C16—C21—C20	121.3 (4)
C8—C7—N1	118.5 (3)	C16—C21—H21	119.4
C12—C7—N1	120.2 (3)	C20—C21—H21	119.4
C9—C8—C7	119.8 (3)	C24—C23—C28	117.5 (3)
C9—C8—H8	120.1	C24—C23—C4	121.3 (3)
C7—C8—H8	120.1	C28—C23—C4	121.2 (3)
C10—C9—C8	118.2 (3)	C25—C24—C23	119.3 (3)
C10—C9—H9	120.9	C25—C24—H24	120.3
C8—C9—H9	120.9	C23—C24—H24	120.3
C11—C10—C9	122.6 (3)	N26—C25—C24	123.7 (3)
C11—C10—N13	118.9 (3)	N26—C25—H25	118.1
C9—C10—N13	118.4 (3)	C24—C25—H25	118.1
C10—C11—C12	119.1 (3)	C27—N26—C25	116.5 (3)
C10—C11—H11	120.5	N26—C27—C28	124.6 (3)
C12—C11—H11	120.5	N26—C27—H27	117.7
C11—C12—C7	118.9 (3)	C28—C27—H27	117.7
C11—C12—H12	120.6	C27—C28—C23	118.4 (3)
C7—C12—H12	120.6	C27—C28—H28	120.8
O15—N13—O14	123.3 (3)	C23—C28—H28	120.8
O15—N13—C10	118.7 (3)		
			
N5—N1—C2—N6	179.8 (3)	C11—C10—N13—O15	177.7 (3)
C7—N1—C2—N6	−3.0 (5)	C9—C10—N13—O15	−5.4 (4)
N5—N1—C2—C3	−0.3 (3)	C11—C10—N13—O14	−4.2 (4)
C7—N1—C2—C3	176.9 (3)	C9—C10—N13—O14	172.8 (3)
N1—C2—C3—C4	−0.6 (3)	C2—C3—C16—C21	59.9 (4)
N6—C2—C3—C4	179.2 (3)	C4—C3—C16—C21	−118.1 (4)
N1—C2—C3—C16	−179.1 (3)	C2—C3—C16—C17	−122.7 (3)
N6—C2—C3—C16	0.8 (5)	C4—C3—C16—C17	59.2 (5)
C2—C3—C4—N5	1.4 (4)	C21—C16—C17—C18	2.1 (4)
C16—C3—C4—N5	179.8 (3)	C3—C16—C17—C18	−175.3 (3)
C2—C3—C4—C23	−175.8 (3)	C16—C17—C18—C19	−1.5 (5)
C16—C3—C4—C23	2.5 (6)	C17—C18—C19—F22	−179.6 (3)
C3—C4—N5—N1	−1.6 (3)	C17—C18—C19—C20	0.3 (5)
C23—C4—N5—N1	176.0 (3)	F22—C19—C20—C21	−179.8 (3)
C2—N1—N5—C4	1.1 (3)	C18—C19—C20—C21	0.3 (5)
C7—N1—N5—C4	−176.4 (3)	C17—C16—C21—C20	−1.5 (5)
C2—N1—C7—C8	139.2 (3)	C3—C16—C21—C20	176.0 (3)
N5—N1—C7—C8	−43.8 (4)	C19—C20—C21—C16	0.3 (5)
C2—N1—C7—C12	−44.6 (5)	N5—C4—C23—C24	25.7 (5)
N5—N1—C7—C12	132.4 (3)	C3—C4—C23—C24	−157.2 (3)
C12—C7—C8—C9	−4.0 (5)	N5—C4—C23—C28	−152.6 (3)
N1—C7—C8—C9	172.2 (3)	C3—C4—C23—C28	24.5 (5)
C7—C8—C9—C10	−0.7 (5)	C28—C23—C24—C25	−0.5 (5)
C8—C9—C10—C11	4.0 (5)	C4—C23—C24—C25	−178.9 (3)
C8—C9—C10—N13	−172.8 (3)	C23—C24—C25—N26	−0.8 (5)
C9—C10—C11—C12	−2.6 (5)	C24—C25—N26—C27	1.1 (5)
N13—C10—C11—C12	174.2 (3)	C25—N26—C27—C28	0.1 (5)
C10—C11—C12—C7	−2.2 (4)	N26—C27—C28—C23	−1.4 (5)
C8—C7—C12—C11	5.4 (5)	C24—C23—C28—C27	1.5 (5)
N1—C7—C12—C11	−170.7 (3)	C4—C23—C28—C27	179.9 (3)

### Hydrogen-bond geometry (Å, º)

**Table:**

*D*—H···*A*	*D*—H	H···*A*	*D*···*A*	*D*—H···*A*
N6—H6*A*···O14^i^	0.91	2.29	3.149 (4)	158
N6—H6*B*···N26^ii^	0.87	2.19	2.985 (3)	151

Symmetry codes: (i) *x*, *y*−1, *z*; (ii) *x*, *y*, *z*+1.
